# New Appliances and Things Medical

**Published:** 1898-04-16

**Authors:** 


### NEW APPLIANCES AND THINGS MEDICAL.

### INSECTICIDES AND VERMIN DESTROYERS.

(J. P. Shorey, 67, Farringdon Road, E.C.)

Samples of these preparations have been sent to us. Peterman's cockroach and beetle food, and the bed-pest destroyer contain no ingredients that would poison children and domestic pets, but are composed of such materials as have invariably been found efficacious for the purposes indicated. The rat and mice food, however, consists of a cereal, containing an admixture of a highly powerful poison, arsenic, in considerable quantity. It is directed that the poison should not be placed where it is likely to come into contact with the family food, and that some responsible person should be in charge of the material. These directions are highly necessary considering that the preparation contains a poison which is included in the Poison Schedule, part 1, of the Pharmacy Act, and, moreover, that it is not coloured in accordance with the stipulations of the Act. It would be most imprudent to keep such a poisonous vermin-destroyer in private households.

